# Supplementary material for: Just Play Cognitive Modern Board and Card Games, It’s Going to Be Good for Your Executive Functions: A Randomized Controlled Trial with Children at Risk of Social Exclusion
Source: Children (Basel). 2023 Aug 31;10(9):1492. doi: 10.3390/children10091492 (PMC10527566; doi:10.3390/children10091492)
Supplement: Supplementary file 1 [file children-10-01492-s001.zip › children-2449051-supplementary.pdf]

Table S1. Scale to assess the satisfaction through the session.

To which game have you played? ____

How many points/cards have you earned at the end of the game? ______________________

Which score do you give to this game? Please, mark one option

1	2	3	4	5	6	7	8	9	10
Very bad									Very good

How have you been playing?

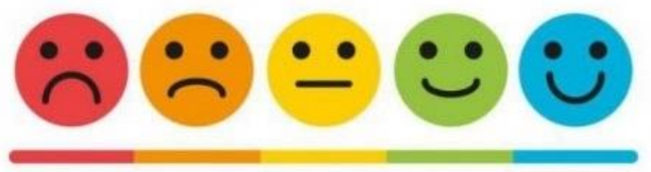

Very bad Bad Regular Good Very good

Table S2. Brief explanation of the games used and justification of the main cognitive process active when playing.

Board game	Description of the game dynamic	Main cognitive process
	GAMES USED IN THE EXPERIMENTAL GROUP	
Bee Alert [2]	A series of colored bees are hidden under their hives. Players must memorize their starting position. Bees, under their hives, move through the game space according to specific cards. When the bees move, the players need to erase from their memory the initial position and update it in the memory	Updating
Jungle Speed [41]	A wooden totem is in the middle of the table. All the cards are distributed to the players. In turns, players face up the cards. When two cards with exactly the same simbol appear, the first person to take the wooden totem wins. The symbols are very similar, so sometimes players need to inhibit the automatic action of taking the totem when they notice that the two symbols are not really the same. In addition, there are some cards that request all the players to do different actions, activating cognitive shifting.	Inhibition and flexibility
Ghost Blitz [42]	Five wooden objects are placed in the middle of the table. All the cards are distributed to the players. In turns, players face up the cards. In each card, two objects with two colours are depicted. However, there are two types of cards. In one type, one of the objects matches the figure and color exactly to one object in the middle of the table. The first person to take the correct object, wins the card. The secon type of card consists in a combination of the shapes and colors that doesn't match any object. But always one of the five objects is not represented in the card, neither by the shape nor by the color. Then, the first player to take this object, wins the card. Then, all the people is switching between the two types of cards. In addition, sometimes the players need to inhibit the automatic action of taking one object when they notice that they are mistaking.	Inhibition and flexbility
Dejà Vu [43]	Different cardboard tokens are laid on the table. One player begin to flip cards, hiding one on the previous one (only one is faced up in each round). Images of the cardboard tokens are depicted in each card (from two to five images). When one player thinks that one object have appeared the second time, he/she takes the token from the center of the table. In this game you need to update constantly the images that are your memory to decide whether it is the first time you see them or is the secon time.	Updating
	GAMES USED IN THE CONTROL GROUP	
Dixit [44]	In each round, one person is a "storyteller". He/she select one card from their hand and, withoug showing the image depicted to the rest of players, he/she makes up a sentence. Then, the other players select one card from their hands that they thinks could fit to the description heard. The storyteller shuffles all the cards and put them face up in a raw and the rest of players try to guess which card whas the chosen one by the storyteller.	Theory of mind

	Depending on the hits and mistakes, all the players score points. To win in this game, every player needs to discern whether the rest of players will select their cards or not without asking directly to them.	
Mixmo [45]	Letter tiles are placed in the middle of the table face down. Each player takes six of them. When the game begins all the players turn their tiles and try to make words linked by one letter. When one player uses all the letter, he/she says Mixmo and every player takes two more tiles. So, the game consists on making words very fast.	Verbal fluency
Story Cubes [46]	There are some dice with figures in their faces. In each player turn, he/she throw all of them and explains a story relating all the dice.	Verbal fluency
Mmm! [47]	A board with 19 pieaces of food of two to four squares are sketched. In each turn, the player roll three dice with the food depicted in each face. In every roll, the player must place at least one die in a food of the board that matches the face of the roll. After three rolls, it the player cannot place the three dice, he/she loose the oportunity of filling the board squares giving a penalty to all the players. In this game is very important to now when is a good moment to take risks and when is better not to reroll.	Decision-making under risk

Note. The main cognitive process active when playing, was estimated before applying the games in the intervention by two senior researchers according to the dynamics of the game.

Table S3. Latent Class Analysis of the evolution in playing each game.

GAMES IN THE EXPERIMENTAL GROUP

Game 1 (Deja-Vu)

	G	loglik	conv	npm	AIC	BIC	SABIC	entropy	ICL	%class1	%class2	%class3
lcga1	1	-348.86	1	3	703.7338	708.8005	699.4127	1.0000000	708.8005	100		
lcga2	2	-298.93	1	6	609.8750	620.0083	601.2327	0.8994997	541.6750	40	60	
lcga3	3	-296.09	1	9	610.1911	625.3910	597.2276	0.7305492	556.1047	35	35	30

	G	loglik	conv	npm	AIC	BIC	SABIC	entropy	ICL	%class1	%class2	%class3
gmm1	1	-306.71	1	4	621.4202	628.1757	615.6587	1.0000000	628.1757	100.0		
gmm2	2	-296.65	1	8	609.3104	622.8215	597.7874	0.7424893	548.9031	55.0	45	
gmm3	3	-295.49	1	12	614.9900	635.2565	597.7053	0.7078822	566.1900	27.5	35	37.5

	G	loglik	conv	npm	AIC	BIC	SABIC	entropy	ICL	%class1	%class2	%class3
gmm1_2	1	-302.08	1	6	616.1763	626.3096	607.5340	1.0000000	626.3096	100.0		
gmm2_2	2	-296.63	1	10	613.2616	630.1504	598.8577	0.7559594	555.9121	55.0	45	
gmm3_2	3	-295.46	1	14	618.9280	642.5724	598.7627	0.7059223	573.5769	27.5	35	37.5

	G	loglik	conv	npm	AIC	BIC	SABIC	entropy	ICL	%class1	%class2	%class3
gmm1_3	1	-306.51	1	5	623.0265	631.4709	615.8246	1.0000000	631.4709	100.0		
gmm2_3	2	-296.51	1	9	611.0383	626.2382	598.0749	0.8228766	549.7733	60.0	40	
gmm3_3	3	-295.25	1	13	616.5031	638.4586	597.7781	0.7361113	569.0132	22.5	40	37.5

Game 2 (Jungle Speed)

	G	loglik	conv	npm	AIC	BIC	SABIC	entropy	ICL	%class1	%class2	%class3
lcga1	1	-279.32	1	3	564.6551	569.8681	560.4735	1.0000000	569.8681	100.00000		
lcga2	2	-262.17	1	6	536.3411	546.7671	527.9779	0.9867162	462.9404	92.85714	7.142857	
lcga3	3	-262.13	1	9	542.2636	557.9026	529.7188	0.9633598	474.9806	92.85714	0.000000	7.142857

	G	loglik	conv	npm	AIC	BIC	SABIC	entropy	ICL	%class1	%class2	%class3
gmm1	1	-272.06	1	4	552.1312	559.0819	546.5557	1.000000	559.0819	100.00000		
gmm2	2	-262.17	1	8	540.3411	554.2424	529.1902	0.986716	470.4158	7.14285	92.85714	
gmm3	3	-1.e+09	4	12	2.00e+09	2.00e+09	2.00e+09	1.000000	2.00e+09	0.00000	0.00000	0

	G	loglik	conv	npm	AIC	BIC	SABIC	entropy	ICL	%class1	%class2	%class3

```
gmm1_2 1   -270.70   1   6 553.4170 563.8430 545.0538 1.0000000 563.8430 100.000000
gmm2_2 2   -262.15   1  10 544.3150 561.6917 530.3764 0.9870903 477.8566   7.142857 92.857143
gmm3_2 3   -262.15   1  14 552.3082 576.6355 532.7941 0.4981886 515.7765  88.095238  7.142857 4.761905
       G   loglik conv npm      AIC      BIC    SABIC   entropy      ICL   %class1  %class2  %class3
gmm1_3 1   -270.60   1   5 551.2151 559.9035 544.2458 1.0000000 559.9035 100.000000
gmm2_3 2   -259.41   1   9 536.8250 552.4640 524.2802 0.9713064 469.4211   7.142857 92.85714
gmm3_3 3   -259.37   1  13 544.7544 567.3441 526.6342 0.4417335 512.8454   7.142857 28.57143 64.28571
```

Game 3 (Bee alert)

```
      G   loglik conv npm      AIC      BIC    SABIC   entropy      ICL %class1 %class2 %class3
lcga1 1 -275.3050    1   3 556.6100 561.6766 552.2888 1.0000000 561.6766   100.0
lcga2 2 -272.7644    1   6 557.5287 567.6620 548.8864 0.5230242 499.6220    27.5    72.5
lcga3 3 -272.7644    1   9 563.5287 578.7287 550.5653 0.2479948 531.3438    70.0     0.0      30
     G        loglik conv npm          AIC          BIC        SABIC   entropy          ICL %class1 %class2 %class3
gmm1 1 -2.749137e+02    1   4 5.578275e+02 5.645830e+02 5.520659e+02 1.0000000 5.645830e+02   100.0
gmm2 2 -2.727644e+02    1   8 5.615287e+02 5.750398e+02 5.500057e+02 0.5230242 5.069998e+02    27.5    72.5
gmm3 3 -1.000000e+09    4  12 2.000000e+09 2.000000e+09 2.000000e+09 1.0000000 2.000000e+09     0.0     0.0       0
       G   loglik conv npm      AIC      BIC    SABIC   entropy      ICL %class1 %class2 %class3
gmm1_2 1 -273.3656    1   6 558.7312 568.8645 550.0889 1.0000000 568.8645   100.0
gmm2_2 2 -272.4872    2  10 564.9743 581.8631 550.5705 0.5851072 512.1929    15.0      85
gmm3_2 3 -271.4678    1  14 570.9356 594.5799 550.7702 0.6279997 528.4358    67.5      25     7.5
       G   loglik conv npm      AIC      BIC    SABIC   entropy      ICL %class1 %class2 %class3
gmm1_3 1 -271.8886    1   5 553.7772 562.2216 546.5753 1.0000000 562.2216   100.0
gmm2_3 2 -269.9264    1   9 557.8529 573.0528 544.8894 0.5242896 504.5760    27.5    72.5
gmm3_3 3 -269.3171    1  13 564.6342 586.5896 545.9092 0.3540472 531.7093    35.0    27.5    37.5
```

Game 4 (Ghost blitz)

```
       G   loglik conv npm      AIC      BIC    SABIC   entropy      ICL %class1 %class2 %class3
lcga1  1 -362.7068    1   3 731.4136 736.4802 727.0924 1.0000000 736.4802   100.0
lcga2  2 -314.7383    1   6 641.4767 651.6100 632.8344 0.8587699 574.7613    70.0    30.0
lcga3  3 -312.5855    1   9 643.1709 658.3708 630.2075 0.6785763 590.6440    37.5    32.5      30
       G   loglik conv npm      AIC      BIC    SABIC   entropy      ICL %class1 %class2 %class3
```

gmm1	1	-327.9895	1	4	663.9790	670.7345	658.2175	1.0000000	670.7345	100		
gmm2	2	-312.7028	1	8	641.4055	654.9166	629.8825	0.8109574	578.7420	30	70.0	
gmm3	3	-311.2305	1	12	646.4610	666.7276	629.1764	0.6527467	599.5928	35	32.5	32.5

	G	loglik	conv	npm	AIC	BIC	SABIC	entropy	ICL	%class1	%class2	%class3
gmm1_2	1	-320.1054	1	6	652.2108	662.3441	643.5685	1.0000000	662.3441	100		
gmm2_2	2	-310.9156	1	10	641.8312	658.7200	627.4274	0.8259024	582.5818	70	30	
gmm3_2	3	-310.4083	1	14	648.8166	672.4609	628.6512	0.7892330	600.0956	20	70	10

	G	loglik	conv	npm	AIC	BIC	SABIC	entropy	ICL	%class1	%class2	%class3
gmm1_3	1	-327.4906	1	5	664.9813	673.4257	657.7793	1.0000000	673.4257	100.0		
gmm2_3	2	-310.0091	1	9	638.0182	653.2182	625.0548	0.8792127	575.6940	67.5	32.5	
gmm3_3	3	-307.4605	1	13	640.9211	662.8765	622.1961	0.7300299	592.6022	22.5	45.0	32.5

GAMES IN THE CONTROL GROUP

Game 1 (Dixit)

	G	loglik	conv	npm	AIC	BIC	SABIC	entropy	ICL	%class1	%class2	%class3
lcga1	1	-394.4369	1	3	794.8738	800.2938	790.8901	1.0000000	800.2938	100.00000		
lcga2	2	-375.1004	1	6	762.2007	773.0407	754.2333	0.7523395	689.4281	26.66667	73.33333	
lcga3	3	-372.2895	1	9	762.5790	778.8390	750.6279	0.6530218	702.3439	20.00000	24.44444	55.55556

	G	loglik	conv	npm	AIC	BIC	SABIC	entropy	ICL	%class1	%class2	%class3
gmm1	1	-378.7174	1	4	765.4348	772.6614	760.1231	1.0000000	772.6614	100.00000		
gmm2	2	-374.1835	1	8	764.3669	778.8202	753.7437	0.5812068	698.7418	73.33333	26.66667	
gmm3	3	-371.0778	1	12	766.1556	787.8356	750.2208	0.6106486	711.9140	20.00000	55.55556	24.44444

	G	loglik	conv	npm	AIC	BIC	SABIC	entropy	ICL	%class1	%class2	%class3
gmm1_2	1	-377.7336	1	6	767.4672	778.3071	759.4997	1.0000000	778.3071	100.00000		
gmm2_2	2	-373.2967	1	10	766.5933	784.6600	753.3143	0.8131777	699.4968	13.33333	86.66667	
gmm3_2	3	-370.9350	1	14	769.8699	795.1632	751.2792	0.6133375	719.1685	20.00000	55.55556	24.44444

	G	loglik	conv	npm	AIC	BIC	SABIC	entropy	ICL	%class1	%class2	%class3
gmm1_3	1	-376.6591	1	5	763.3183	772.3516	756.6788	1.0000000	772.3516	100.000000		
gmm2_3	2	-372.2798	1	9	762.5596	778.8195	750.6084	0.6929753	697.2812	28.888889	71.11111	
gmm3_3	3	-367.4487	1	13	760.8975	784.3841	743.6347	0.7572255	704.1616	8.888889	40.00000	51.11111

Game 2 (Mmm!)

	G	loglik	conv	npm	AIC	BIC	SABIC	entropy	ICL	%class1	%class2	%class3
lcga1	1	-485.2884	1	3	976.5767	981.8603	972.4625	1.0000000	981.8603	100.00000		
lcga2	2	-408.4995	1	6	828.9990	839.5662	820.7707	0.9687003	754.2294	69.76744	30.23256	
lcga3	3	-398.8696	1	9	815.7393	831.5901	803.3968	0.9351875	748.4206	13.95349	69.76744	16.27907

	G	loglik	conv	npm	AIC	BIC	SABIC	entropy	ICL	%class1	%class2	%class3
gmm1	1	-419.3984	1	4	846.7967	853.8415	841.3111	1.0000000	853.8415	100.00000		
**gmm2**	**2**	**-399.5052**	**1**	**8**	**815.0103**	**829.0999**	**804.0392**	**0.9337421**	**744.6925**	**69.76744**	**30.232558**	
gmm3	3	-394.6102	1	12	813.2205	834.3549	796.7638	0.9391178	750.5145	62.79070	2.325581	34.88372

	G	loglik	conv	npm	AIC	BIC	SABIC	entropy	ICL	%class1	%class2	%class3
gmm1_2	1	-412.1845	1	6	836.3689	846.9361	828.1406	1.0000000	846.9361	100.0000		
gmm2_2	2	-397.5927	1	10	815.1855	832.7975	801.4716	0.9116509	748.6559	62.7907	37.209302	
gmm3_2	3	-394.3040	1	14	816.6079	841.2647	797.4084	0.9395800	757.3940	62.7907	2.325581	34.88372

	G	loglik	conv	npm	AIC	BIC	SABIC	entropy	ICL	%class1	%class2	%class3
gmm1_3	1	-418.7350	1	5	847.4701	856.2761	840.6131	1.0000000	856.2761	100.000000		
gmm2_3	2	-400.5383	1	9	819.0766	834.9274	806.7341	0.9422624	750.1420	30.232558	69.76744	
gmm3_3	3	-396.6852	1	13	819.3703	842.2659	801.5422	0.9448621	757.9302	4.651163	67.44186	27.90698

Game 3 (Story cubes)

	G	loglik	conv	npm	AIC	BIC	SABIC	entropy	ICL	%class1	%class2	%class3
lcga1	1	-20.60549	1	3	47.210997	52.56356	43.162760	1.0000000	52.56357	100.00000		
lcga2	2	0.88635	1	6	10.227289	20.93242	2.130814	0.8600284	-64.00215	22.72727	77.27273	
**lcga3**	**3**	**13.52423**	**1**	**9**	**-9.048474**	**7.00923**	**-21.193186**	**0.8033956**	**-74.07344**	**50.00000**	**27.27273**	**22.72727**

	G	loglik	conv	npm	AIC	BIC	SABIC	entropy	ICL	%class1	%class2	%class3
gmm1	1	-5.347778	1	4	18.695556	25.83231	13.297906	1.0000000	25.83231	100.00000		
gmm2	2	1.477188	1	8	13.045624	27.31914	2.250324	0.6329425	-50.31119	22.72727	77.27273	
gmm3	3	13.524237	1	12	-3.048474	18.36180	-19.241423	0.8033964	-62.72090	22.72727	50.00000	27.27273

	G	loglik	conv	npm	AIC	BIC	SABIC	entropy	ICL	%class1	%class2	%class3
gmm1_2	1	-3.067831	1	6	18.135661	28.84080	10.03919	1.0000000	28.84080	100.00000		
gmm2_2	2	11.044135	2	10	-2.088270	15.75363	-15.58239	0.9245108	-70.98336	77.27273	22.72727	
gmm3_2	3	13.524237	1	14	0.951525	25.93018	-17.94025	0.8033962	-55.15251	50.00000	22.72727	27.27273

	G	loglik	conv	npm	AIC	BIC	SABIC	entropy	ICL	%class1	%class2	%class3

```
gmm1_3 1 -1.636040    1   5 13.272080 22.19303   6.525018 1.0000000  22.19303 100.00000
gmm2_3 2  5.917644    1   9  6.164711 22.22242  -5.980000 0.8714145 -63.00365  77.27273 22.72727
gmm3_3 3 14.073291    1  13 -2.146583 21.04788 -19.688945 0.7789466 -58.74891  27.27273 22.72727     50
```

Game 4 (Mixmo)

```
      G    loglik conv npm      AIC      BIC    SABIC   entropy      ICL   %class1  %class2 %class3
lcga1  1 -327.3445    1   3 660.6890 666.1090 656.7052 1.0000000 666.1090 100.00000
lcga2  2 -308.9178    1   6 629.8357 640.6757 621.8683 0.9734609 551.0493  91.11111 8.888889
lcga3  3 -303.2983    1   9 624.5966 640.8565 612.6454 0.8544106 555.7768  11.11111 8.888889     80
      G    loglik conv npm      AIC      BIC    SABIC   entropy      ICL    %class1  %class2  %class3
gmm1   1 -315.0450    1   4 638.0900 645.3166 632.7783 1.0000000 645.3166 100.000000
gmm2   2 -305.5450    1   8 627.0900 641.5433 616.4668 0.9699371 552.3662   8.888889 91.11111
gmm3   3 -303.2983    2  12 630.5966 652.2765 614.6617 0.8544099 567.1969   8.888889 80.00000 11.11111
      G    loglik conv npm      AIC      BIC    SABIC   entropy      ICL   %class1   %class2  %class3
gmm1_2 1 -312.3766    1   6 636.7532 647.5932 628.7858 1.0000000 647.5932 100.00000
gmm2_2 2 -306.5310    1  10 633.0619 651.1285 619.7828 0.4284326 576.6864  75.55556 24.444444
gmm3_2 3 -303.2983    1  14 634.5966 659.8898 616.0059 0.8544101 574.8102  80.00000  8.888889 11.11111
      G    loglik conv npm      AIC      BIC    SABIC   entropy      ICL   %class1   %class2  %class3
gmm1_3 1 -312.8637    1   5 635.7273 644.7606 629.0878 1.0000000 644.7606 100.00000
gmm2_3 2 -303.2856    1   9 624.5713 640.8312 612.6201 0.9532432 551.7100  91.11111  8.888889
gmm3_3 3 -301.0909    1  13 628.1817 651.6683 610.9190 0.8570276 566.6134  77.77778 13.333333 8.888889
```

Note. Selected models are marked in red.

Table S4. Modulation of the evolution through games in the effects of the cognitive intervention.

Game	Cognitive process	F	p
Deja-vu	KTT - Linguistic Working Memory	.8934	.348
	KTT - Visuospatial Working Memory	.017	.898
	5D – Inhibition	2.616	.115
	5D – Flexibility	.4275	.518
Ghost blitz	KTT - Linguistic Working Memory	.799	.374
	KTT - Visuospatial Working Memory	.262	.613
	5D – Inhibition	.408	.527
	5D – Flexibility	**4.721**	**.036**
Mmm!	KTT - Linguistic Working Memory	.951	.338
	KTT - Visuospatial Working Memory	.988	.332
	5D – Inhibition	.127	.724
	5D – Flexibility	.739	.397
Story cubes	KTT - Linguistic Working Memory	1.435	.255
	KTT - Visuospatial Working Memory	.371	.695
	5D – Inhibition	.982	.386
	5D – Flexibility	.188	.829
Mixmo	KTT - Linguistic Working Memory	.208	.650
	KTT - Visuospatial Working Memory	**5.788**	**.025**
	5D – Inhibition	.294	.592
	5D – Flexibility	.013	.912

Note. The table only depicts the specific results of the interaction between the intraindividual factor (pre-post) and the group of the game.
